# The Capsule of Cryptococcus neoformans Modulates Phagosomal pH through Its Acid-Base Properties

**DOI:** 10.1128/mSphere.00437-18

**Published:** 2018-10-24

**Authors:** Carlos M. De Leon-Rodriguez, Man Shun Fu, M. Osman Çorbali, Radames J. B. Cordero, Arturo Casadevall

**Affiliations:** a Department of Molecular Microbiology and Immunology, Johns Hopkins Bloomberg School of Public Health, Baltimore, Maryland, USA; Duke University Medical Center

**Keywords:** *Cryptococcus*, capsule, pH, phagosome

## Abstract

Phagosomal acidification is a critical cellular mechanism for the inhibition and killing of ingested microbes by phagocytic cells. The acidic environment activates microbicidal proteins and creates an unfavorable environment for the growth of many microbes. Consequently, numerous pathogenic microbes have developed strategies for countering phagosomal acidification through various mechanisms that include interference with phagosome maturation. The human-pathogenic fungus Cryptococcus neoformans resides in acidic phagosomes after macrophage ingestion that actually provides a favorable environment for replication, since the fungus replicates faster at acidic pH. We hypothesized that the glucuronic acid residues in the capsular polysaccharide had the capacity to affect phagosomal acidity through their acid-base properties. A ratiometric fluorescence comparison of imaged phagosomes containing C. neoformans to phagosomes containing beads showed that the latter were significantly more acidic. Similarly, phagosomes containing nonencapsulated C. neoformans cells were more acidic than those containing encapsulated cells. Acid-base titrations of isolated C. neoformans polysaccharide revealed that it behaves as a weak acid with maximal buffering capacity around pH 4 to 5. We interpret these results as indicating that the glucuronic acid residues in the C. neoformans capsular polysaccharide can buffer phagosomal acidification. Interference with phagosomal acidification represents a new function for the cryptococcal capsule in virulence and suggests the importance of considering the acid-base properties of microbial capsules in the host-microbe interaction for other microbes with charged residues in their capsules.

**IMPORTANCE** Cryptococcus neoformans is the causative agent of cryptococcosis, a devastating fungal disease that affects thousands of individuals worldwide. This fungus has the capacity to survive inside phagocytic cells, which contributes to persistence of infection and dissemination. One of the major antimicrobial mechanisms of host phagocytes is to acidify the phagosomal compartment after ingestion of microbes. This study shows that the capsule of C. neoformans can interfere with full phagosomal acidification by serving as a buffer.

## Abstract (precis)

Cryptococcus neoformans is the causative agent of cryptococcosis, a devastating fungal disease that affects thousands of individuals worldwide. This fungus has the capacity to survive inside phagocytic cells, which contributes to persistence of infection and dissemination. One of the major antimicrobial mechanisms of host phagocytes is to acidify the phagosomal compartment after ingestion of microbes. This study shows that the capsule of C. neoformans can interfere with full phagosomal acidification by serving as a buffer.

## INTRODUCTION

Ingestion of microbes by phagocytic cells results in the formation of a new organelle called the phagolysosome, a membrane-bound compartment where microorganisms are subjected to a variety of antimicrobial compounds such as oxidative radicals and microbicidal proteins. The process of phagolysosome formation results from a complex cellular choreography that includes phagosome acidification, resulting in microbial inhibition by creating an unfavorable environment and the activation of various microbicidal compounds. Consequently, diverse pathogenic microbes have developed mechanisms to interfere with phagosomal acidification. For example, the bacterium Mycobacterium tuberculosis (1), the fungus Histoplasma capsulatum (2), and the parasite Leishmania donovani (3) each avoid phagosomal acidification by interfering with the process of phagosome maturation, reducing the presence of the vesicular proton-ATPase from phagosomes. Hence, modulation of phagosomal acidification by microbes ingested by phagocytic cells and the mechanisms for such effects are topics of great interest and research activity in the field of microbial pathogenesis research.

Cryptococcus neoformans is a facultative intracellular pathogenic yeast (4) that is a major cause of meningoencephalitis in individuals with impaired immunity (5). In contrast to many other facultative intracellular pathogens, this fungus resides in an acidic phagosome after ingestion by macrophages (6). Despite residing in an acidic phagosome, there is evidence that C. neoformans modulates some aspects of phagosomal maturation, including full phagosomal acidification, although the mechanisms for this effect have not been fully elucidated (7, 8). In fact, for C. neoformans, acidification has been viewed as favoring intracellular growth, since this fungus replicates faster in acidic environments (9). Its survival inside the phagosome is believed to result from its ability to withstand oxidative bursts (10), damage the phagosomal membranes (11), and damage critical host cell homeostasis (12) rather than interference with phagosomal maturation, although the relative contributions to the overall outcome of intracellular survival remain to be determined.

Recently, we reported a new role for C. neoformans urease in modulating phagosomal pH (13). Urease-positive C. neoformans strains hydrolyzed urea to ammonia, resulting in pleiotropic changes to the cryptococcal macrophage interaction that included higher phagosomal pH, delayed intracellular growth, and enhanced nonlytic exocytosis (13). C. neoformans is unusual among intracellular pathogens in that it grows faster at lower pH, resulting in faster replication inside phagolysosomes than in the extracellular medium (9). Loss of phagosomal integrity is associated with reduced acidity in that compartment and the triggering of macrophage death (14). Hence, the extent of phagosomal acidification is an important variable, which can favor the microbe or the host cell depending on the state of the interaction (13, 14).

One of the most striking characteristics of C. neoformans as a pathogenic microbe is that it is surrounded by a large polysaccharide capsule that is a critical determinant of virulence (15). The capsule functions in virulence by interfering with phagocytosis and immune responses (15, 16). The capsule is also thought to play a major role in intracellular survival by quenching free radical fluxes in the phagosome (10). The major capsular polysaccharide is glucuronoxylomannan (GXM), which is composed of a mannose backbone with xylose and glucuronic acid substitutions (17). The presence of glucuronic acid residues in cryptococcal polysaccharide imparts a negative charge to the capsule (18) that is believed to contribute to protection against phagocytosis. In addition, those glucuronic acid residues can be anticipated to impart considerable acid-base properties to the cryptococcal GXM. In our recent study on the role of phagosomal membrane integrity, we observed that even though apoptotic cells had higher phagolysosomal pH, loss of membrane integrity was not associated with complete loss of acidity, which we hypothesized was due to the acid-based properties of the capsule (14). In contrast, for Candida albicans, which lacks a polysaccharide capsule and hence has no comparable buffering capacity, phagosome permeabilization resulted in luminal alkalinization (19). In this study, we formally tested that hypothesis and present evidence that the capsule of C. neoformans interferes with full phagosome acidification. These findings establish a new mechanism for microbial modulation of phagosomal pH and imply a new role for the capsule in cryptococcal virulence.

## RESULTS

### pH of phagosomes containing beads and C. neoformans.

**FIG 1:**
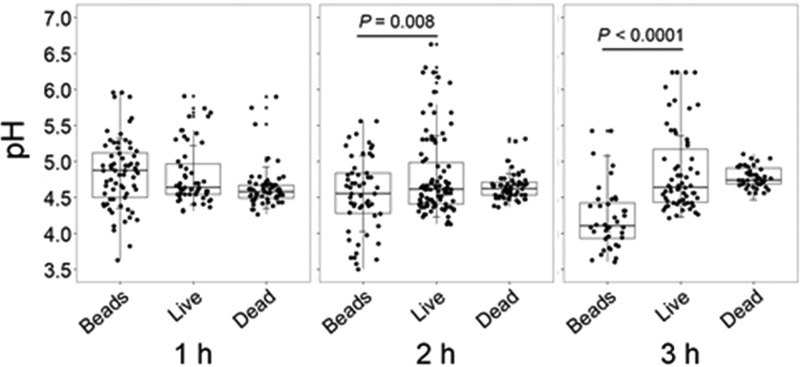
pH of phagosomes containing beads or heat-killed or live C. neoformans. Phagolysosomal pH was measured by using Oregon green dual-excitation ratio fluorescence imaging at the indicated time points. Each dot represents the pH of an individual phagolysosome. Data are from one representative experiment. Comparable results were obtained from two additional independent experiments. *P* values were calculated by one-way ANOVA with Tukey’s multiple-comparison test.

Ingested C. neoformans resides in a mature acidic phagosome (6). However, the extent to which C. neoformans modulates the pH of the cryptococcal phagosome is unknown. A comparison of the pH of phagosomes containing inert beads with phagosomes containing C. neoformans cells showed that the latter were significantly less acidic (Fig. 1). On average, the pH of phagosomes containing inert beads was 4.22 ± 0.45 (*n* = 40) at 3 h, which corresponded to a 0.65 pH unit difference (*P < *0.0001 by one-way ANOVA and Tukey’s multiple-comparison test). To ascertain whether this higher pH was the result of active pH modulation by C. neoformans, we compared the pH of phagosomes containing live and dead C. neoformans cells. Comparison of the average pH in phagosomes containing live and dead C. neoformans cells revealed average values of 4.87 ± 0.58 (*n* = 62) and 4.78 ± 0.14 (*n* = 43) at 3 h, respectively (*P  = *0.638 by one-way ANOVA and Tukey’s multiple-comparison test). Phagosomes containing live and dead cells had comparable pHs to those having live cells, suggesting that the pH modulation in the phagosome is not the result of secretion of basic compounds by C. neoformans (Fig. 1).

### The phagosomal pH of encapsulated cells is higher than that of nonencapsulated cells.

**FIG 2:**
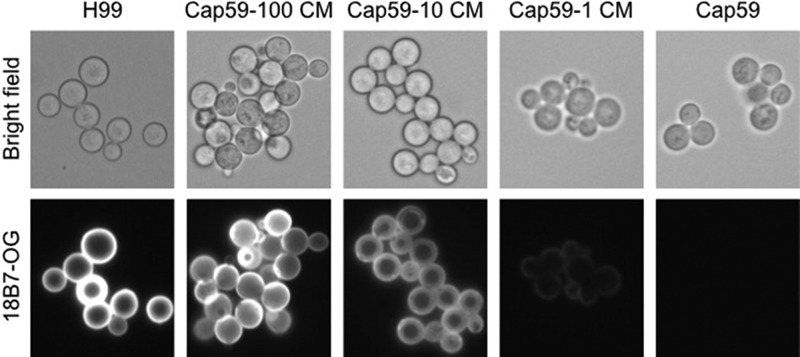
Binding of capsular polysaccharide to the nonencapsulated C. neoformans *cap59* strain. Capsule material (CM) release into the media by C. neoformans was incubated with different concentrations of the *cap59* strain to form a proto-capsule around these cells. Subsequently, the cells were incubated with the monoclonal antibody 18B7 previously conjugated with Oregon green (18B7-OG). Bright-field (top) and immunofluorescence (bottom) images are shown of C. neoformans H99 and the acapsular *cap59* strain incubated with 100, 10, or 1 μl of CM and *cap59* strain alone. The magnification for this figure is 40×.

**FIG 3:**
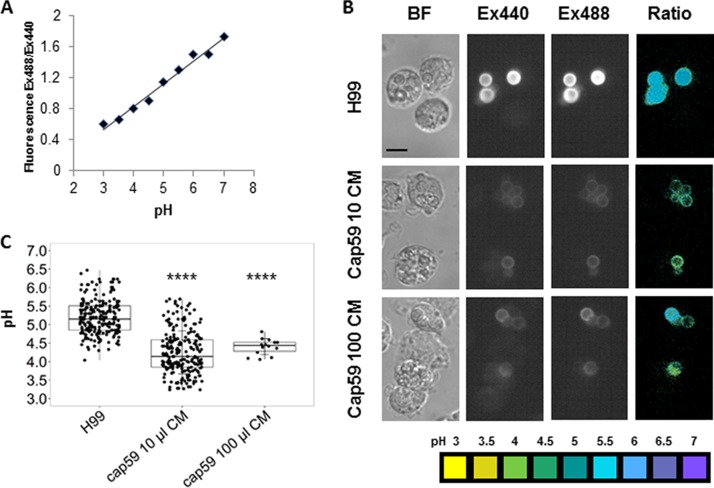
pH measurement of phagolysosome-containing encapsulated and proto-encapsulated C. neoformans. Macrophages were infected with H99 and *cap59* strains previously incubated with 100 and 10 µl of conditioned media (CM) to form a proto-capsule. Opsonization was antibody mediated by using monoclonal antibody 18B7 conjugated to Oregon green. Phagolysosomal pH was measured by using dual-excitation ratio fluorescence imaging. (A) Representative standard curve of mean fluorescence excitation ratio (488-nm excitation/440-nm excitation: 520-nm emission) of Oregon green cryptococcus-loaded macrophages with phagolysosomes equalized in calibration buffers at pH 3 to pH 7. (B) Representative images showing bright-field (BF) microscopy of macrophages containing cryptococcal cells, fluorescence images taken at 440-nm excitation (Ex440) and 488-nm excitation (Ex488), and pseudocolor images of the 488ex/440ex ratio with pH color scale displayed at the bottom of the panel. Scale bar, 10 μm. (C) Box plot with dots representing pH of individual phagolysosomes. The pH value was 5.19 in H99- containing phagolysosomes, which was less acidic than phagosomes containing *cap59* strain incubated with 100 and 10 μl of CM, showing pH values of 4.40 and 4.25, respectively. Fewer data points are plotted for the *cap59* 100-μl CM conditions relative to the other conditions because their tendency to aggregate in that experiment but the numbers were sufficient for high statistical significance. One-way ANOVA with Dunnett’s multiple-comparison test was conducted. ****, *P*  < 0.0001 (Tukey’s multiple comparison).

To test the hypothesis that pH modulation was the result of the acid-base properties of C. neoformans, we sought to compare the phagosomal pH for encapsulated and nonencapsulated cells. However, this presented the practical problem that nonencapsulated cells could not be opsonized through the FcR, since they lacked a capsule that would bind GXM-binding antibody. Opsonizing encapsulated cells with antibody and nonencapsulated cells with complement was not considered acceptable, since the two opsonins are very different. We tried to label cryptococcal cells with EZ-Link NHS-biotin and Oregon green 488-conjugated NeutrAvidin, and phagocytosis was performed using guinea pig complement as an opsonin. However, the signal of Oregon green was not stable in the phagosome, and after 24 h, it was lost completely, which we attribute to dye degradation from a combination of the low phagosomal pH and the oxidative burst (20). We have also tried to measure the phagolysosomal pH with heat-killed cryptococcal cells labeled with NHS-biotin, but the labeling did not work with heat-killed cells. Hence, we resorted to coating nonencapsulated cells with encapsulated C. neoformans conditioned media, which results in the attachment of soluble polysaccharide to the surfaces of nonencapsulated cells to create a proto-capsule that would allow antibody-mediated opsonization (Fig. 2). The phagosomal pH of naturally encapsulated C. neoformans cells was significantly higher than that of nonencapsulated cells containing an artificial capsule (Fig. 3).

### Acid-base properties of glucuronic acid and GXM.

**FIG 4:**
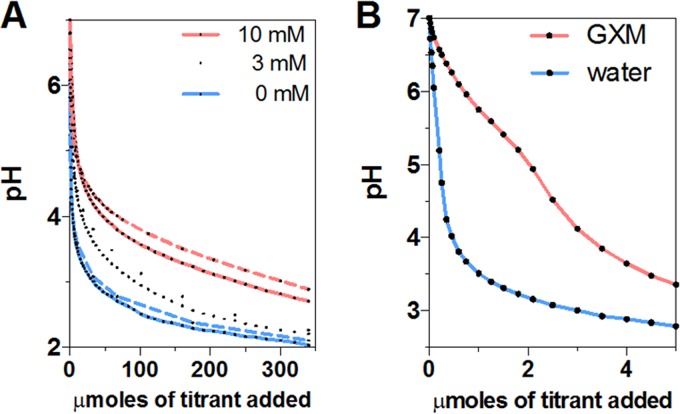
Titration of sodium D-glucuronate and GXM against HCl. (A) Changes in pH of sodium D-glucuronate solutions at 3 and 10 mM as a function of the number of micromoles of titrant added. Initially, sodium D-glucuronate solutions show a rapid pH change by acidic titration. Near the pKa level (pH ∼3.28, it shows the best buffering capacity compared to pure water. Theoretical curves (dashed lines) for glucuronic acid also reveal a similar tendency of rapid pH change and buffering capacity. (B) Change in pH of GXM solution as a function of the number of micromoles of titrant added. GXM provides a substantial amount of buffering capacity around pH 5 compared to ultrapure water.

Glucuronic acid is an organic weak acid with a relatively high pKa. We titrated a sodium salt of glucuronic acid (sodium D-glucuronate) with HCl and calculated a pKa in the range of 2.5 to 3.11 at the beginning of our titrations (corresponding to 0.23 to 20 µmol of titrant) (Fig. 4A). These values are comparable to the reported pKa values of 2.9 (21), 2.8 to 2.9 from ^13^C-nuclear magnetic spectroscopy (22), and 3.28 measured from standard acid-based titrations (23). Consequently, the presence of glucuronic acid in solution confers considerable buffering capacity such that for a 10 mM solution to change from pH 7 to pH 4, it requires almost 10 times the acid required to achieve the same pH reduction as in a pure water solution. Similarly, the presence of GXM in water provided considerable buffering capacity at around pH 5, which is close to the final pH in cryptococcal phagosomes (Fig. 4B). Considering the known polyelectrolyte nature and polydispersity of GXM preparations in terms of molecular mass (24, 25), together with the mild inflection point, it is problematic to determine a pKa value for GXM.

## DISCUSSION

Many pathogenic microbes express polysaccharide capsules that are essential for virulence. Microbial capsules are directly antiphagocytic, and efficient ingestion of encapsulated microbes by phagocytic cells usually requires antibody- or complement-derived opsonins. However, many microbial capsules are composed of polysaccharides that are poorly immunogenic that often fail to induce strong responses. Although microbial capsules are generally thought to contribute to virulence by resisting ingestion and killing by host phagocytic cells, there is evidence that capsules mediate other functions that contribute to pathogenesis and that some of these effects are mediated by capsule ionic charge. For instance, negatively charged capsular polysaccharides of Gram-negative bacteria can bind cationic microbicidal peptides and protect bacterial cells (26). On the other hand, the positively charged modifications in *Streptococcus pneumoniae* capsular polysaccharide can predispose bacterial cells to enhanced killing by alpha-defensins (27). Among *Cryptococcus* spp., a comparison of glucuronic acid residue content among a nonpathogenic species and pathogenic species revealed that the latter had higher content of this charged residue (28). Hence, while there is considerable evidence that microbial surface charge can play an important role in pathogenesis through a variety of mechanisms, their role in phagosomal pH modulation has not been investigated.

It is axiomatic that microbial capsules containing weak acid and basic residues will exhibit acid-base properties that are reflective of the capacity of these residues to function as proton donors and acceptors. In this regard, the cryptococcal capsule is composed of a repeating mannose triad, each of which includes one glucuronic acid residue, that in turn confers upon the polysaccharide and the resulting capsule a negative charge (18). Our results confirm the theoretical deduction that the C. neoformans capsular GXM buffers the phagolysosomal pH. While titration of the sodium salt of glucuronic acid (sodium D-glucuronate) shows a rapid initial decrease in pH during titration, GXM provides a buffer effect around pH 5. This is not surprising, since the pKa of glucuronic acid is 3.28 and a solution of sodium D-glucuronate will exert its best buffering effect near the pKa. However, with readily available acidic and basic residues of glucuronic acid (A^−^ and HA) covalently bound to the GXM polysaccharide backbone, these residues could experience different electronic milieu that could modify their ionic properties. Furthermore, GXM molecules are large and structurally complex, and consequently, not all glucuronic acids may be equally exposed to the solvent and in a position to donate or accept hydronium ions equally. Polyelectrolytes can manifest complex acid-base properties and the pKa values of the monomer and polymer can differ (29, 30). Despite these differences, GXM retains considerable weak acid properties that would confer a maximum buffering capacity at the pH range of approximately 4 to 5, which corresponds to the optimal pH for C. neoformans growth (13).

Ingested dead encapsulated C. neoformans cells resided in phagosomes that had a higher pH than phagosomes containing inert beads. This suggested that C. neoformans cells contained anionic groups that could buffer hydronium ions in the phagosome, resulting in a higher pH. To ascertain the contribution of the capsule to this effect, we compared the phagosomal pH of C. neoformans encapsulated cells to acapsular cells coated with a proto-capsule that would allow both to be opsonized with IgG1 through the same Fc receptors. We recognize that adding GXM to acapsular cells to create a proto-capsule that permitted antibody-mediated opsonization meant that we undermined any comparison involving GXM acid-base effects, since this maneuver introduced some polysaccharide into the phagosome. We reasoned that this handicap was outweighed by the fact that we could not be certain that other methods of opsonization would result in comparable phagosomes and the fact that natural capsules are much larger than artificial capsules meant that there would still be much more polysaccharide in encapsulated cryptococcal phagosomes. Despite this handicap, we were able to measure a difference in phagosomal pH between encapsulated and acapsular strains consistent with a strong acid buffering capacity by the polysaccharide capsule.

In summary, the presence of glucuronic acid residues in the C. neoformans capsule makes the polysaccharide a weak acid capable of modulating pH in the phagosome. Our experimental observations are consistent with the expected acid-base properties of the capsule based on its sugar residue composition. The fact that the polysaccharide capsule of C. neoformans is large brings considerable GXM mass into the phagosome with the potential to mediate considerable buffering capacity. Given that C. neoformans has optimal growth rate at acidic pHs (13), the acid-base properties of the capsule can be expected to promote fungal cell survival in the phagosome by its buffering capacity during conditions of both phagosomal acidification and phagosomal membrane leakage. This mechanism for phagosomal pH modulation based on acid-base properties is different from other intracellular pathogens that modulate pH by interfering with phagosome maturation. Our observations suggest that other microbes with charged microbial capsules could also modulate phagosomal acidification through their acid-based electrolyte properties.

## MATERIALS AND METHODS

### Yeast culture.

C. neoformans serotype A strain H99 and the acapsular mutant *cap59* were used for all experiments. Cells were grown from frozen stocks in Sabouraud dextrose liquid medium at 30ºC under agitation (180 rpm) for 2 days.

### Coating of acapsular mutant with GXM.

For the formation of the proto-capsule, we follow previously published methods (31). Briefly, the supernatant of an overnight culture of C. neoformans H99 was cleared by centrifugation and filtered using a 0.8-µm syringe filter. An overnight culture of 1 × 10^7^ cells/ml *cap59* acapsular strain was then incubated with 100, 10, or 1 µl of H99 cleared supernatant (conditioned media) in a total volume of 1 ml medium with rotation for 1 h at room temperature. Images were acquired using an Olympus AX70 microscope (Olympus, Center Valley, PA) with 40× objective to visualize the formation of the proto-capsule, which was labeled by Oregon green 488 conjugated 18B7 monoclonal antibody.

### Measurement of phagosomal pH.

Phagolysosomal pH was measured using ratiometric fluorescence imaging involving the use of pH-sensitive probe Oregon green 488. Oregon green 488 was first conjugated to monoclonal antibody 18B7 using Oregon green 488 protein labeling kit (Molecular Probes, Eugene, OR) as described previously (13). The Oregon green 488 dye has a succinimidyl ester moiety that reacts with primary amines of proteins to form stable dye-protein conjugates. The labeling procedure was performed according to the manufacturer’s instruction. BMDM were plated (4 × 10^5^ cells/well) on 24-well plate with 12-mm circular coverslip coated with 100 µg/ml poly-D-lysine. Cells were cultured with completed BMEM medium containing 0.5 µg/ml LPS and 100 U/ml IFN-γ and then incubated at 37°C with 9.5% CO_2_ overnight. For infection, H99 and *cap59* strains (8 × 10^6^ cells/ml) were incubated with 10 µg/ml Oregon green conjugated mAb18B7 for 15 min. Macrophages were then infected with Oregon green-conjugated 18B7-opsonized yeast in 4 × 10^5^ cells per well. Cells were centrifuged immediately at 270 ×  *g* for 1 min, and the cells were incubated at 37°C for 10 min to allow phagocytosis. Extracellular cryptococcal cells or beads were removed by washing three times with fresh medium. Samples on coverslip were collected at 24 h after phagocytosis by washing twice with prewarmed HBSS. Annexin V Alexa Fluor 555 staining was performed per the manufacturer’s instructions (Invitrogen, Carlsbad, CA). The coverslip was then placed upside down on a MatTek petri dish (35 mm; 10-mm diameter microwell; MatTek, Ashland, MA) with the Annexin V binding buffer in the microwell. Images were taken by using an Olympus AX70 microscope (Olympus, Center Valley, PA) with 40× objective with two excitation wavelengths of 440 nm and 488 nm for Oregon green and wavelength of 550 mm for Annexin V and bright-field microscopy. Images were acquired and analyzed using MetaFluor Fluorescence Ratio Imaging Software (Molecular Devices, Downingtown, PA). Relative phagolysosomal pH was determined based on the ratio of 488 nm/440 nm. The relative pH was converted to absolute pH by obtaining the standard curve in which the images are taken as described above, but the intracellular pH of macrophage was equilibrated by adding 10 µM nigericin in pH buffer (140 mM KCl, 1 mM MgCl_2,_ 1 mM CaCl_2,_ 5 mM glucose, and appropriate buffer ≤ pH 5.0: acetate-acetic acid; pH 5.5 to 6.5: MES; ≥pH 7.0: HEPES. Desired pH values were adjusted using either 1 M KOH or 1 M HCl). Buffers were used at pH 3 to 7.5 using 0.5-pH unit increments.

### Biotinylation of cells.

Approximately 1 × 10^6^ cryptococcal cells were biotinylated using EZ Link-sulfo-NHS-biotin (catalog no. 21217; Thermo Fisher Scientific, Rockford, IL). Overnight cultures were washed three times with PBS (pH 8.0) and diluted in PBS (pH 8.0) to 1 × 10^6^ cells/ml. EZ Link-sulfo-NHS-biotin (2 mM) was added to the cell samples and incubated for 30 min at room temperature. Cells were then washed three times with PBS with 100 mM glycine to remove excess biotin reagent and by-product. After biotinylation, cells were labeled with 5 µg/ml Oregon green conjugate of NeutrAvidin biotin-binding protein (catalog no. A6374; Thermo Fisher Scientific) with rotation for 1 h at room temperature.

### GXM isolation.

Soluble GXM was obtained from culture supernatants of encapsulated cells by ultrafiltration (32, 33). Briefly, culture supernatants were collected by centrifugation (6,000 *× g*, 15 min, 4°C) and filtered using 0.22-µm vacuum-driven disposable bottle-top filter (MilliPore) to ensure clearing of cells and other large debris. The cleared supernatant was ultrafiltered sequentially in an Amicon ultrafiltration cell (Millipore, Danvers, MA) using membranes of 100- and 10-kDa nominal molecular weight limits. After filtrating using a 100-kDa membrane, the flowthrough was again filtered through a 10-kDa membrane. On each filtration step, GXM can be recovered from the surfaces of membranes in the form of a viscous gel. This process yields GXM fractions of >100 kDa and 100 to 10 kDa that were then dialyzed against ultrapure water, lyophilized, and store until use.

### Acid-based titrations.

Forty milliliter solutions of 3 and 10 mM sodium D-glucuronate (catalog no. G8645; Sigma) were titrated against 0.1 M HCl in 40-ml glass beakers. Since the pH of ultrapure water was acidic at ∼pH 5, it was adjusted to pH 7 using NaOH before preparation of the glucoronate solutions. GXM solutions were prepared by dissolving the lyophilized GXM fraction 10 to 100 kDa in ultrapure water at 1 mg/ml. Since GXM molecules exhibit wide size distributions, from 1 to >100 kDa, we used the 10- to 100-kDa fraction to narrow the range of molecular mass. The total monosaccharide concentrations in GXM solutions were determined by the Dubois method (also known as the phenol-sulfuric acid assay) (34). After the phenol sulfuric assay, GXM solutions were diluted to 2.4 mM total monosaccharide concentration, and 3-ml volumes were titrated against 0.01 M HCl in 10-ml glass beakers. Titrations were conducted in beakers placed inside a water bath equilibrated at a temperature of 20°C with constant stirring. Changes in pH were recorded using an Accumet pH meter.

### Calculation of theoretical acid-base titration curve.

To calculate the theoretical acid-base titration curve for sodium D-glucuronate (*NaA*), we assumed that positive charges and negative charges are equal in an ideal solution: [Na^+^] + [H^+^] = [OH^−^] + [A^−^] + [CI^−^], such that NaA↔Na^+^ + A^−^ and NaA + HCl↔HA + NaCl. Such that [Na^+^] = [A^−^] + [HA]. Also, C_HA_ = [HA] + [A^−^] and C_HCI_ = [CI^−^], since *HCl* totally dissolves. Hence, the equation becomes

$$C_{HA}+\left[H^{+}\right]=\left[{OH}^{-}\right]+\left[A^{-}\right]+C_{HCl}$$

At this point, we can turn this equation into a third-degree polynomial, with [H^+^] being the unknown,

$$x^{3}+\left(\frac{n}{V_{initial}+V_{HCl}}+k_{a}-C_{HCl}\right)x^{2}-\left(k_{a}C_{HCl}+k_{water}\right)x-k_{water}k_{a}=0$$

while pKa = 3.28 (glucuronic acid), *k*_water_ = 0.681 × 10^−14^ at 20°C, and *n* is the initial amount of moles of sodium D-glucuronate we use for our solution. The positive root of this third-degree polynomial provided us the theoretical [H^+^] value after a certain number of moles of acid was added.

### Statistical analysis.

All statistical analyses were performed by using one-way ANOVA, followed by Tukey’s or Dunnett’s multiple-comparison test.
